# Melting Point Depression of Poly(ethylene oxide)-Poly(propylene oxide)-Poly(ethylene oxide) Triblock Polymers in Supercritical Carbon Dioxide in the Presence of Menthol as a Solid Co-Plasticiser

**DOI:** 10.3390/polym14142825

**Published:** 2022-07-11

**Authors:** Vivek Trivedi, Adejumoke Lara Ajiboye, Nichola J. Coleman, Ruchir Bhomia, Marion Bascougnano

**Affiliations:** 1 Medway School of Pharmacy, University of Kent, Central Avenue, Chatham Maritime, Kent ME4 4TB, UK; l.ajiboye@kent.ac.uk; 2 Department of Pharmaceutical, Chemical and Environmental Science, University of Greenwich, Central Avenue, Chatham Maritime, Kent ME4 4TB, UK; n.coleman@gre.ac.uk (N.J.C.); marion.bascougnano19@gmail.com (M.B.); 3 Procter & Gamble, 452 Basingstoke Road, Reading RG2 0RX, UK; ruchirbhomia@gmail.com

**Keywords:** melting point depression, triblock polymers, supercritical carbon dioxide, solid co-plasticiser

## Abstract

The melting behaviour of the triblock polymers, Pluronic F38, F68, F77, F108, and F127, was investigated in pressurised CO_2_ and in the presence of menthol. The melting points of the polymers combined with 0, 10, 25, and 50 wt% of menthol were studied at atmospheric pressure and compared with those at 10 and 20 MPa in supercritical carbon dioxide (scCO_2_). The highest melting point depressions of 16.8 ± 0.5 °C and 29.0 ± 0.3 °C were observed at 10 and 20 MPa, respectively. The melting point of triblock polymers in pressurised CO_2_ was found to be dependent on molecular weight, poly(propylene oxide) (PPO) content, and menthol percentage. The melting point of most of the polymers studied in this work can be reduced to room temperature, which can be pivotal to the formulation development of thermolabile substances using these polymers.

## 1. Introduction

**Figure 1:**
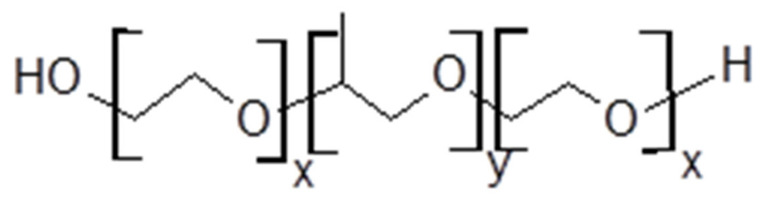
Generalised structure of a PEO-PPO-PEO triblock polymer (x and y represent the length of PEO and PPO blocks, respectively).

Triblock copolymers commonly, known as poloxamers or Pluronics, consist of hydrophilic poly(ethylene oxide) (PEO) and hydrophobic poly(propylene oxide) (PPO) segments which are arranged in an A-B-A structure as shown in Figure 1.

These polymers are synthesised by sequential polymerisation by building the PPO block first followed by the addition of PEO chains at both ends of the polymer [1]. The lengths of the PPO and PEO blocks can be tailored to fine-tune the properties of the polymers, including crystallinity conferred by the PEO block, melting point (Tm), hydrophilic–lipophilic balance (HLB), and solubility [2]. The triblock polymers studied in this work are commercially available as Pluronic^®^ F38, F68, F77, F108, and F127. These are solid at room temperature and their average molecular weight, T_m_, and the number of PEO/PPO units are presented in Table 1.

The interaction of scCO_2_ with polymers is well documented where the dissolution of CO_2_ in the polymer is known to cause a depression in T_m_ and/or glass transition temperature (T_g_) [3,4]. The knowledge of the phase behaviour of polymers in pressurised systems is of great importance, as this may enable the processing of formulations at lower temperatures to avoid the decomposition of thermolabile drugs during formulation. Investigations of the solubility or miscibility of a single solute in a binary system in contact with a single supercritical fluid (SCF) are widely reported [5,6,7,8,9,10]. For example, T_m_ depression in polymers caused by the dissolution of CO_2_ is very well known and has been widely reported [3,4,11,12,13,14]. This phenomenon and the extent of T_m_ reduction are dependent on numerous factors including the actual melting temperature, crystal density, amorphous phase density, enthalpy of fusion, repeat unit molecular weight, and presence of CO_2_-philic moieties. However, interactions between a solute, excipient(s), and SCF in multicomponent systems are less frequently documented in the literature, especially with regard to the alteration of physical properties such as the T_m_ and/or T_g_ by their possible action as a co-plasticiser.

**Figure 2:**
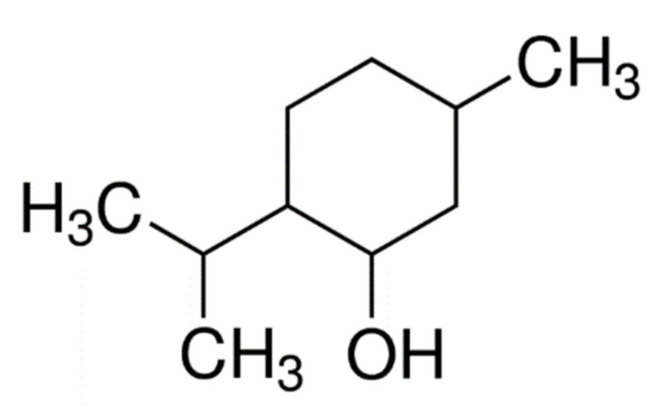
Chemical structure of menthol.

The present study investigates the effect of menthol (Figure 2) on the melting behaviour of these triblock polymers in scCO_2_. Menthol (C_6_H_9_OHCH_3_C_3_H_7_), a naturally occurring saturated secondary alcohol, is one of the major components in peppermint oil [15].

There are numerous reports on the use of menthol as a co-solvent for particle engineering and to improve solubility in SCF processes as listed in Table 2.

Menthol is a favoured plasticiser for SCF processes because it has excellent solubility in scCO_2_ along with sufficiently high vapour pressure for facile removal by sublimation at the end of the process [28]. Menthol is a non-toxic and inexpensive chemical with ‘GRAS’ (generally recognised as safe) status and is regularly used in cosmetic, food, pharmaceutical, and consumer health products (e.g., mouthwashes, sprays, comestibles, and topical formulations) [29].

Pluronics have found numerous applications in the pharmaceutical industry due to their non-ionic, amphiphilic, and non-toxic nature; ability to form micelles; thermoresponsive gelling properties; and excellent biocompatibility and biodegradability [30,31]. They are commonly used in drug solubilisation, gene/therapeutic delivery, diagnostics, and tissue engineering applications. Their properties and various applications in pharmaceutical formulations and drug delivery are comprehensively discussed in a number of recently published reviews [31,32,33,34,35,36]. Pluronics are also commonly used in the preparation of solid dispersions (SDs) to improve the dissolution rate of poorly soluble drugs. The SDs can be prepared using several techniques, but the heat fusion method is still one of the most common and simple methods, which requires heating the Pluronic–drug mixture above the polymer’s melting point [35,36,37,38,39,40,41,42]. The temperatures involved in the preparation of these SDs using Pluronics range from 63 to 160 °C. The importance of the current study is in establishing if the working temperature for Pluronics in applications such as in the preparation of SDs can be significantly lowered. The principal objective of this work was to investigate whether menthol could be used to depress the T_m_ of a range of triblock polymers in scCO_2_ to room temperature (25 °C). The significance of the presented information relates to particle engineering and in the formulation development of thermolabile substances using PEO-PPO-PEO block copolymers.

## 2. Experimental Section

### 2.1. Materials

Pluronic^®^ F38 and F77 were kindly donated by BASF (Ludwigshafen, Germany). Other polymers (Pluronic^®^ F68, F108, and F127) and menthol (>99%) were purchased from Sigma Aldrich (Gillingham, UK). Liquid carbon dioxide was obtained from BOC Ltd. (Guildford, UK) with a purity of 99.9%. All reagents used in this study were of analytical grade and used without further purification.

### 2.2. Methods

Mixtures of poloxamer and menthol were prepared by manually homogenising 10, 25, and 50 wt% menthol with each polymer in a pestle and mortar. Samples were initially analysed by differential scanning calorimetry at atmospheric pressure and then at 10 and 20 MPa in scCO_2_ using a supercritical phase monitor (SPM). Menthol content was incrementally increased until a T_m_ of 25 °C (i.e., room temperature) was achieved.

#### 2.2.1. Determination of Melting Temperature of Pluronics and Mixtures in scCO_2_

**Figure 3:**
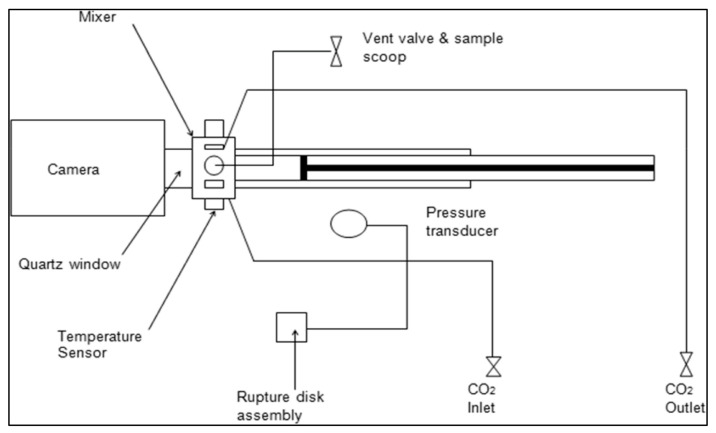
Schematics of supercritical phase monitor.

The melting point depression in scCO_2_ was determined using a supercritical phase monitor (Supercritical Fluid Technologies (SFT) Inc., Newark, DE, USA). The phase monitor (Figure 3) contains a manually controlled syringe pump attached to a 30 mL pressure vessel, a CCD camera with a fibre optic light source to allow clear viewing of the vessel’s interior, and a magnetic stirrer for effective mixing. The heating of the vessel is controlled by an internal resistance thermometer up to a maximum temperature of 150 °C.

The instrument was calibrated using a naphthalene standard prior to the experiments. For T_m_ determination, a melting point capillary was filled with 1–3 mg of sample, and CO_2_ was introduced into the vessel to achieve the desired pressure which was then kept constant during the experiment by manually rotating the piston. The temperature under constant pressure was increased in increments of 0.2 °C until the complete melt was observed. The melting of the polymer and the polymer/menthol mixtures was monitored through a quartz window via a camera attached to the vessel. The melting point depression (ΔT) was calculated by subtracting the T_m_ of the polymer in the presence of menthol at atmospheric (0.1 MPa) or higher pressures (10 or 20 MPa) in scCO_2_ from their actual melting temperature. The experiments were conducted in triplicate and each repeat was performed on a freshly prepared Pluronic–menthol mixture to establish sample homogeneity. No further experiments were conducted on the polymer–menthol mixtures below 25 °C, primarily as the major objective of this study was to determine if the T_m_ of Pluronics could be lowered to ‘room temperature’ using a scCO_2_–menthol binary mixture. Moreover, it would also have been technically impossible to conduct experiments below room temperature due to the instrument’s lack of cooling capability.

The menthol in samples after processing was completely removed prior to any analysis. All processed samples were removed from the vessel and were maintained for 1 week at ambient temperature and pressure. Thereafter, polymers were kept under a vacuum (5 mbar) for 4 h to ensure the complete removal of menthol from the samples. The complete removal of menthol was determined via weight loss, i.e., until samples achieved constant weight and no more weight reduction was observed with the longer or higher vacuum.

#### 2.2.2. Differential Scanning Calorimetry (DSC)

Melting point determinations of the samples with menthol at atmospheric pressure (0.1 MPa) and after processing were performed using a DSC-1 calorimeter (Mettler-Toledo). Between 5 and 8 mg of each sample was sealed in a standard aluminium pan and DSC thermograms were recorded from 25 to 100 °C at a heating rate of 10 °C min^−1^ with an equilibration time of 120 s.

#### 2.2.3. X-ray Diffraction

The diffractograms of unprocessed and scCO_2_-processed polymers were collected using a Bruker D8 Advance (Bruker, Germany) diffractometer in theta–theta reflection mode with a copper anode. Each sample was scanned from 2° to 60° at a step size of 0.02° in the 2θ range. Data collection and interpretations were performed using DiffracPlus and the EVA V.14 program, respectively.

## 3. Results and Discussion

The T_m_ of triblock polymers and their menthol mixtures at atmospheric pressure (0.1 MPa) was determined using DSC and compared to understand the plasticisation effect of menthol. Table 3 presents the T_m_ of individual polymers and mixtures with 10, 25, and 50 wt% of menthol at 0.1, 10, and 20 MPa.

It is evident from the data in Table 3 that menthol is a suitable plasticiser for Pluronics and is capable of depressing the T_m_ by its interposition between the polymer chains resulting in the weakening of the attractive forces between adjacent polymer molecules. The depression in T_m_ of the Pluronics with varying menthol content was found to be between 6 and 9 °C depending on the polymer. The lowering of the T_m_ was found to be highest for F108, the Pluronic with the greatest PEO content and highest average molecular weight, indicating that the interaction of menthol with Pluronics is dependent on molecular weight and PEO/PPO ratio.

**Figure 4:**
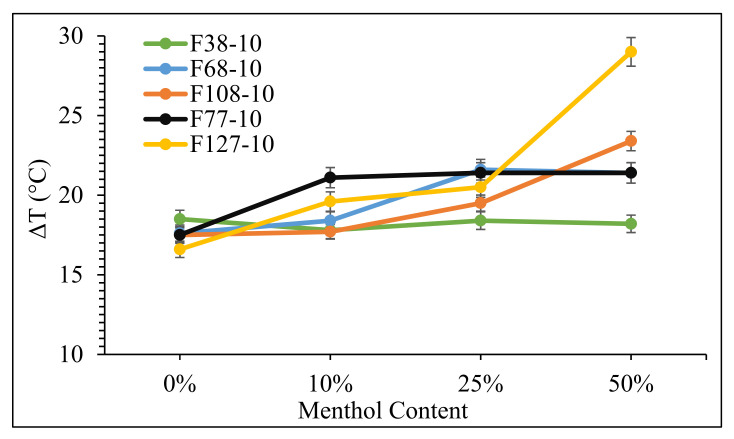
Melting point depression (ΔT) in Pluronics at 10 MPa in scCO_2_ as a function of menthol content.

The addition of menthol led to a further reduction in T_m_ in the range of 17 to 29 °C for these polymers in the presence of scCO_2_. The melting point depression obtained at 10 MPa with increasing menthol content is presented in Figure 4.

In general, there was an increase in the extent of T_m_ depression for all polymers which was highest for F127 with 50 wt% of menthol at 10 MPa. This is attributed to the comparatively high polarity of the scCO_2_–menthol solution compared with that of CO_2_ alone and the resulting ease with which the menthol and CO_2_ molecules interact with the polymer [5]. Notably, both Pluronic F77 and F127 melted at room temperature (25 °C) with 25% and 50 wt% additions of menthol, respectively. Polymers with comparatively greater PPO fractions (F77 and F127 with ~30% PPO) showed a higher depression in T_m_ with increasing menthol content in scCO_2_ in comparison to those with lower PPO fractions of ~20% (F38, F68, and F108).

**Figure 5:**
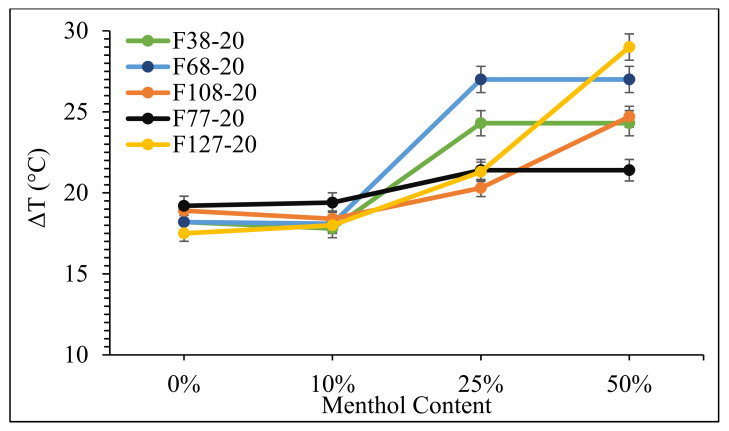
Melting point depression (ΔT) in Pluronics at 20 MPa in scCO_2_ as a function of menthol content.

The density of the scCO_2_ phase can also play an important role with respect to the T_m_ depression phenomena. The trend in T_m_ reduction at 20 MPa was similar to that observed at 10 MPa where a higher depression was obtained with increasing menthol content. Interestingly, an increase in pressure from 10 to 20 MPa alone did not have a significant impact on the melting temperatures of these polymers, as reported in a previous study by Bhomia et al. [3]. However, the combined effect of a higher density of scCO_2_ and the presence of a co-plasticiser on the melting behaviour of Pluronics is evident from the data presented in Figure 5.

The addition of 10 wt% menthol showed minimal effect on the T_m_ of polymers but mixtures with 25 wt% menthol lowered the T_m_ of F38, F68, and F77 to 25 °C at 20 MPa. A further increase in menthol to 50 wt% also resulted in the lowering of the T_m_ of F108 and F127 to 33 and 25 °C, respectively. Hence, with the exception of F108, the melting points of all polymers studied in this work could be reduced to room temperature in the presence of menthol and in scCO_2_.

A correlation between the PPO content, molecular weight, and PEO/PPO ratio to the extent of T_m_ depression could be seen in the data obtained in this study. The polymers with a higher number of PPO units showed a higher ΔT at 10 MPa, which could especially be seen with 50 wt% menthol (F38 > F68 ≈ F77 > F108 > F127). The depression in melting temperature of Pluronics was still dependent on the PPO content at 20 MPa, but an increase in pressure had a significantly greater impact on the melting of polymers with 14 and 30 PPO units (F38 and F68, respectively). This can be attributed to several factors, including the polarity of the scCO_2_–menthol binary mixture, improved accessibility of the CO_2_-philic functional groups at 20 MPa, the density of the medium, and the lower molecular weight of these polymers.

A summary of the lowest T_m_ obtained for all five Pluronics at respective pressure and menthol content is presented in Table 4.

The reduction in T_m_ in compressed CO_2_ is not a hydrostatic pressure effect but a colligative property which is enhanced by the incorporation and accessibility of CO_2_-philic functionalities, e.g., ether linkages, carbonyl, and fluro groups. It is known that the gas molecules preferentially penetrate the amorphous regions of a semicrystalline polymer to facilitate Lewis acid/base interactions between the polymer and CO_2_ [12,43]. This leads to the depression in T_m_ due to the reduction in the chemical potential of the amorphous regions, driving the morphology to a more amorphous state and therefore reducing the crystallinity [11,12,13,44,45]. Furthermore, scCO_2_ is also known to act as a molecular lubricant by weakly solvating the molecular segments of the polymer, thus assisting in the reduction of T_m_. Menthol and scCO_2_ are individually good plasticisers for Pluronics, but this research has demonstrated that a mixture of both clearly improves their interaction with the polymer, resulting in a higher T_m_ depression. It is conjectured that the addition of menthol and its impact on the overall polarity of the fluid medium may be responsible for this effect.

The plasticisation effect of menthol alone was generally higher for Pluronics containing 80% PEO (F38, F68, and F108), which was in contrast to the results obtained with the binary mixture of menthol and scCO_2_. F77 and F127 with 70% PEO showed a sharp decrease in T_m_ even at 10 MPa and melted at 25 °C in the presence of 25% and 50 wt% menthol, respectively. The effect of pressure was particularly evident on polymers with lower PPO content, where a pressure increase to 20 MPa resulted in the lowering of the T_m_ of F38 and F68 to 25 °C with 25 wt% menthol. The T_m_ of the ‘largest’ polymer (F108) in the study was lowered by ~25 °C in the presence of 50 wt% menthol but could not be decreased to room temperature as could the rest of the Pluronics. The effect of pressure could be explained by the improved rate of diffusion of the plasticisers in the polymer matrix. In view of the dynamic solvation–desolvation between the plasticiser molecules and the polymer chains, the higher the diffusion rate, the greater the efficiency of the compound as a plasticiser. Moreover, an increase in pressure does not only increase the density of scCO_2_ at a given temperature but also improves the solubility of menthol; hence, an improvement in plasticisation efficiency of this binary mixture can be expected for the same group of polymers. However, interactions between scCO_2_ and polymers are complicated where numerous factors (i.e., temperature, pressure, co-solvent/plasticiser) can have a simultaneous effect on their phase behaviour. Menthol is an excellent choice for a co-plasticiser with scCO_2_ due to its non-toxic and GRAS status.

**Figure 6:**
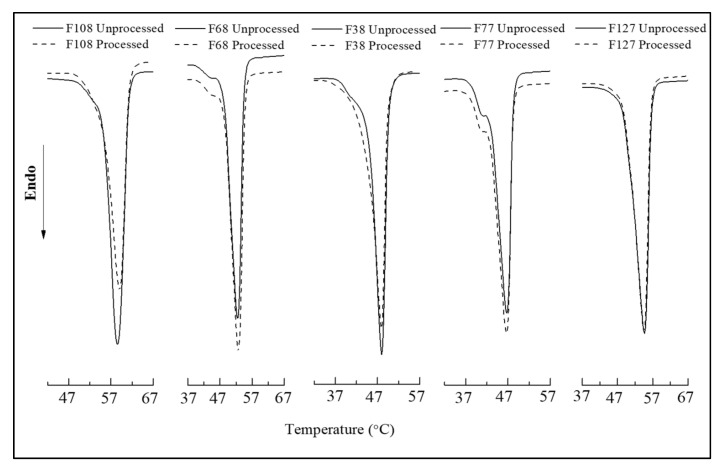
DSC thermograms of Pluronics before and after processing.

The use of plasticisers to reduce the T_m_ of polymers is very common, but it is usually impossible to remove the plasticiser from the matrix after processing. Menthol can be easily removed after the scCO_2_ treatment from the mixture due to its low vapour pressure, resulting in the final product without the presence of residual plasticisers after processing [11]. DSC and XRD analyses were performed to establish this and to determine if the processing at stated conditions had any impact on the polymer. The samples prepared for these analyses were at the pressure and temperatures where the lowest T_m_ was obtained in this study. Figure 6 presents the DSC thermograms of all five Pluronics before and after processing with scCO_2_.

The thermograms of unprocessed and processed Pluronics resulted in a similar melting onset and peaks for all, indicating that the processing of these polymers at the stated conditions had no impact on their thermal properties. Few of the thermograms had shoulders in the peaks, which could be due to the presence of admixtures of PPO homopolymer within the block copolymers, and the differences in the peak height can be attributed to the sample size [3,46].

**Figure 7:**
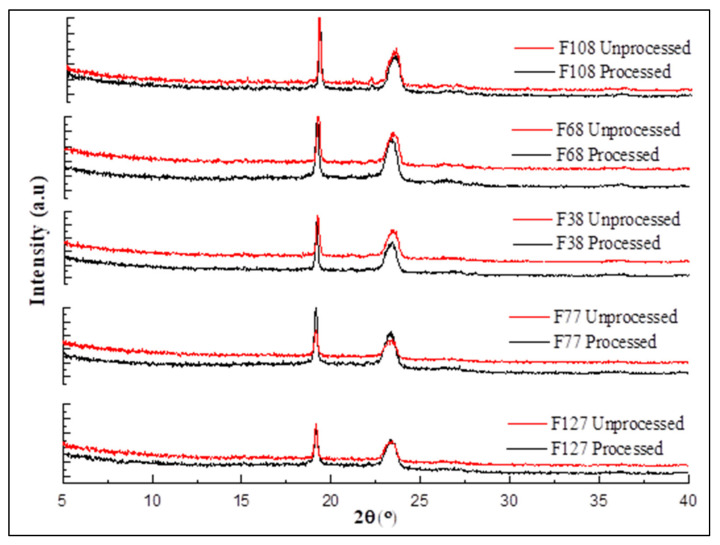
XRD diffractograms of Pluronics before and after processing. Pluronics are semi-crystalline polymers due to the presence of crystalline PEO and amorphous PPO fractions in the structure. The diffractograms of all Pluronics (processed and unprocessed) were identical with peaks at 19 and 23° 2θ originating from the PEO fraction. Similar to DSC, this also confirms that the CO_2_ processing did not cause any changes to the crystal structure of these polymers and indicates the absence of menthol in the sample.

XRD diffractograms of the processed and unprocessed samples of polymers are presented in Figure 7.

## 4. Conclusions

Additions of menthol in the presence of compressed scCO_2_ were observed to increase the interaction between the fluid phase and the Pluronics. In this study, T_m_ depressions in the range 16.8 to 29.0 °C were observed for the polymers. The extent of T_m_ depression was found to be dependent on PPO content, molecular weight, menthol content, and pressure. In general, increases in menthol content led to higher depressions in T_m_ at both 10 and 20 MPa. Also, there was a direct correlation between the PPO content and melting behaviour, where higher PPO-containing polymers showed a greater reduction in T_m_. The increase in pressure to 20 MPa had a significant impact on the depression in melting temperature of Pluronics with lower PPO. This study clearly shows that menthol is an appropriate co-plasticiser for Pluronics that can enable the processing of these polymers near room temperature at comparatively low pressures.

## Figures and Tables

**Table 1:** Properties of PEO-PPO-PEO block copolymers.

Polymer (Pluronic^®^)	Molecular Weight (g·mol^−1^)	T_m_ (°C)	Weight PEO	Weight PPO	PEO Units	PPO Units	PEO:PPO (Units)
F38	4600	48	3680	920	84	16	5.3:1.0
F68	8400	52	6720	1680	152	30	5.2:1.0
F108	14,600	57	11,680	2920	266	50	5.3:1.0
F77	6600	48	4620	1980	106	34	3.1:1.0
F127	12,600	56	8820	3780	202	65	3.1:1.0

**Table 2:** Examples of the application of menthol as a solid co-solvent with scCO_2_.

API	P (MPa)	T (°C)	Purpose	Outcome	Ref.
Megestrol acetate	15–25	40–60	Size reduction	103–515 nm	[16]
Clobetasol propionate	20–26	70–110	Size reduction	95–319 nm	[17]
Phenytoin	9.6–19.6	45	Size reduction	75–120 nm	[18]
Beclomethasone dipropionate	20–26	70–110	Size reduction	65–294 nm	[19]
Letrozole	12–36	45–75	Size reduction	19–260 nm	[20]
Aprepitant	12–33	35–65	Size reduction	85–523 nm	[21]
Sertraline hydrochloride	12–30	35–65	Solubility improvement in scCO_2_	59-fold increase	[22]
Tolbutamide	15–20	35–45	Size reduction	2.1–2.9 μm	[23]
Ketoconazole	12–30	35–65	Solubility improvement in scCO_2_	62-fold increase	[24]
Acetaminophen	10–25	40–70	Solubility improvement in scCO_2_	8-fold increase	[25]
Clozapine Lamotrigine	12.3–33.6	40–50	Solubility improvement in scCO_2_	56-fold increase 8-fold increase	[26]
Aspirin	7.3–8.5	30–70	Size reduction	0.17–6.61 μm	[27]
Griseofulvin	19.6	40	Size reduction	150–155 nm	[15]

**Table 3:** Melting points of Pluronics and polymer/menthol mixtures at 0.1, 10, and 20 MPa.

Pressure (MPa)	Menthol (wt%)	Melting Point of Pluronics (°C)				
		F38	F68	F108	F77	F127
0.1	0	49.4 ± 0.1	52.1 ± 0.2	57.8 ± 0.1	46.5 ± 0.1	54.1 ± 0.2
	10	47.1 ± 0.1	50.6 ± 0.3	54.1 ± 0.3	46.6 ± 0.2	53.1 ± 0.3
	25	44.1 ± 0.2	47.1 ± 0.2	51.1 ± 0.2	43.6 ± 0.4	48.6 ± 0.2
	50	43.2 ± 0.2	44.9 ± 0.1	49.1 ± 0.4	41.6 ± 0.2	46.1 ± 0.2
10	0	30.9 ± 0.6	34.5 ± 0.5	40.3 ± 0.5	29.0 ± 0.3	37.5 ± 0.7
	10	31.7 ± 0.8	33.7 ± 1.3	40.1 ± 0.3	25.4 ± 2.3	34.5 ± 0.3
	25	31.0 ± 0.7	30.5 ± 0.7	38.3 ± 0.4	25.0 ± 0.2 *	33.6 ± 1.3
	50	31.4 ± 1.1	30.7 ± 0.8	34.4 ± 1.0	25.0 ± 0.3 *	25.0 ± 0.2 *
20	0	31.2 ± 0.6	33.9 ± 0.2	38.9 ± 0.3	27.3 ± 0.2	36.6 ± 0.8
	10	32.6 ± 1.1	33.9 ± 1.1	40.1 ± 0.4	27.1 ± 0.5	36.1 ± 0.9
	25	25.0 ± 0.2 *	25.1 ± 0.2 *	37.5 ± 1.6	25.0 ± 0.2 *	32.8 ± 0.7
	50	25.0 ± 0.1 *	25.0 ± 0.4 *	33.1 ± 1.2	25.1 ± 0.2 *	25.0 ± 0.2

* T_m_ of 25 °C was already obtained with lower menthol content or at lower pressures.

**Table 4:** Lowest T_m_ of Pluronics in scCO_2_.

Polymer	Pressure (MPa)	Menthol (wt%)	Melting Point (°C)
F38	20	25	25
F68	20	25	25.1
F108	20	50	33.1
F77	10	25	25
F127	20	50	25.1

## Data Availability

Data are contained within the article.
